# The Principles and Practice of Medicine

**Published:** 1849-01

**Authors:** 


### REVIEWS.

Art. III.—*The Principles and Practice of Medicine.* By John W. Hood, M. D. Philadelphia: Thomas, Copperthwaite, &c, 1848. 8vo. pp. 263.

In these latter days "book-making" has become altogether the rage, and almost every one possesses the ambition of authorship. A prolific press hourly teems with the produce of the busy brains of the votaries of science, and volume follows volume with amazing rapidity.

These differ widely, of course, in value and importance. Some are "filled to the brim and running over" with matter at once original and useful; others are only a remoulding of old ideas and a reiteration of established doctrines and truths; while others still broach wild and startling theories having really no foundation, save in the fantastic brains of the authors, who seem possessed of some unlucky inspiration,

> "Which makes them, though it were in spite
> Of nature, and their stars, to write."

To which one of these classes this work most appropriately belongs, we now propose to inquire. We can best assist our readers in deciding, by giving a few extracts taken almost at random from its pages.

The style, we must premise, is rough and harsh in the extreme; but while we consider it very desirable that the diction of every book should be pleasant, not to say elegant, we are aware that this is not one of the essentials. We often see, in our intercourse with the world, the kindest heart and the soundest head concealed under the roughest exterior; and in like manner, we cannot always estimate the real value of an author's materials by the homely garb in which they are clothed; and this homeliness we are more willing to overlook in this case, inasmuch as the writer lays no claim to anything but a diligent study of nature, and untiring researches in "Clinical Medicine" for the past thirty years.

The opinions of an individual who has been unceasingly engaged in studying pathology by post-mortem examinations, for nearly the third of a century, if they do not carry with them some degree of conviction, are at least, entitled to a fair and candid hearing. Dr. Hood has arrived at conclusions differing widely from the views, so far as we know, of all his professional brethren.

We intend, at this time, not formally to review his work, but rather, as stated above, to give a few extracts, which will serve as an outline of his notions in regard to the causes and treatment of many of the "ills that flesh is heir to."

According then to Dr. Hood, *displacemeant of the abdominal viscera*, should be ranked as *one* of the most frequent, if not *the most frequent* of causes predisposing or exciting, of disease and death.

That we may not be accused of misrepresenting the author in any way, we give the following sentences.

"These *displacements*" (i. e. of the mesentery) "*with those of the organs of the abdominal viscera*, I am fully convinced, from various dissections, are the source of *a numerous class of diseases*."—p. 11.

Again, we are told that the sigmoid flexure of the colon becomes *displaced and descends into the pelvic cavity where it becomes "the cause of hemorhoids."* * * * "This sigmoid flexure of the colon has been, and in my opinion may be considered one of the principal causes of prolapsus uteri."—p. 16. * * * "Is it not strange that so much attention and laborious research should have been expended upon the cause of diseases originating in the abdominal cavity without even a reference to *the gravitation of the viscera of the abdomen and its fluids?*"—p. 18.

*Chronic diseases of the viscera of the abdomen.*—"For illustration, I have grouped together the following diseases for a summary consideration, viz:—Constipation, dyspepsia, chronic diarrhœa, gastritis, enteritis, peritonitis, gastro-enteritis, hepatitis, splenitis, and bilious colic, and predicate the opinion that *the most frequent source of all those diseases is misplacement or subsidence of the abdominal viscera.*"—p. 30. And then on the next page, he relates how he saw, on one occasion, a case of bilious colic which had resisted all the ordinary means and threatened to carry off the sufferer, cured by hanging him up by the heels: whereupon as if by magic "a quantity of gas escaped—a free alvine discharge was had from the bowels, and in twenty minutes the patient was entirely relieved."

But the list by no means ends here. See what he says of *strumous habit.* "*The bowels being permitted to gravitate*, is one of the principal exciting causes of the disease, and to a large class of chronic diseases, is a predisposing cause in some and and an exciting one in others."—p. 71.

*Fever.*—"This being clear it will be seen, *that the tendency of the viscera and fluids to gravitate*, with the altered respiratory movements, are fruitful predisposing and exciting causes of febrile action."—p. 105. "In the laborious research after causes, *the liability of the viscera of the abdomen and fluids to gravitate*, with their influence upon the respiratory movements, upon the blood, and upon the general system, have been misapprehended by the profession."—p. 107. "In my clinical pursuits I have observed *many cases of fever produced by the displaced condition of the small intestines and transverse colon.*"—p. 20.

*Cholera.*—"Bandages and supporters to prevent *gravitation of the viscera* and to assist the capillary vessels in returning the blood to the heart," are recommended as most valuable adjuncts in the treatment of this disease.—p. 167.

*Hemorrhoids.*—"Hence I am inclined to the belief that *the prolapsed condition of the sigmoid flexure of the colon is the primary cause of this painful malady.*"—p. 203.

Here we see the author gravely advocating the doctrine that constipation, dyspepsia, chronic diarrhœa, gastritis, enteritis, peritonitis, gastro-enteritis, hepatitis, splenitis, bilious colic, hemorrhoids, fever, prolapsus uteri, cholera, and strumous habit, have all, in the great majority of cases, for their primary and fundamental cause, *a prolapsed or displaced condition of some one or more of the abdominal organs.* After reading this terrible catalogue, there are few of our readers, we imagine, who will not join with the doctor in exclaiming, "I have been *astonished* at the number of diseases originating from displaced organs!"

For our own part we see no reason why the list may not with equal propriety be extended a little farther and take in erysipelas, cancer, dropsy, epilepsy, hydrothorax, pleurisy, pneumonia, hydrocele, orchitis, eczema, corns, bunions, ulcers, varicocele, &c., &c.

Entertaining views of this kind in relation to the *cause* of so many diseases, of course the *treatment* employed by the author will vary in some important particulars from the commonly accredited practice. Regarding a mechanical displacement of the bowels as the active agent in producing the malady, he sees that all the drugs from every quarter of the world, from the north and the south, the east and west, are insufficient of themselves to effect a cure; that no remedy or combination of remedies directed in all the Pharmacopœias of

> "Every kindred tongue and tribe
> On this terrestrial ball"

will be able to return the stray bowels to their "native home." Accordingly, in addition to the treatment usually directed, he insists on hanging up the patient by the heels, and urges the importance, in a therapeutical point of view, of "supporters," "bandages," "rollers," and "pledgets" applied to the surface of the abdomen.

Thus—"In ordinary constipation," says he, "*I invert the body or elevate the hips occasionally*, *apply a supporter*, direct vegetable diet with bran bread, stewed fruits, &c. and regular and punctual attempts at stool, at a certain hour each day, with gentle exercise, aperients, alteratives," &c.—p. 45–6.

"Dyspepsia * * * is also to be relieved by mild aperients, diet, and *the application of suitable supporters or suspensory jackets.* But before the remedies are applied, the patient should *invert the body* for the purpose of restoring the organs to the place where nature intended they should perform their functions. If confined to the recumbent position, the object may be accomplished by elevating the hips and flexing the thighs upon the body for *fifteen minutes* every twenty-four hours or by *inverting the body one minute*"—p. 46. "In diarrhœa from constipation and gravitated viscera or fluids, *reverse the body* or elevate the hips so as to replace the bowels, and *apply a supporter* to prevent gravition."—p. 50.

When it depends upon acute or chronic inflammation of the intestines he directs the usual remedies.

"When they fail," says he, "I have been able to control the disease by a *flannel roller and pledgets.*"—p. 50.

"In acute or chronic inflammation," he repeats again, "I have found the *roller* an efficient agent."—p. 50. "*Bandages are as strongly indicated*," pursues he, "in inflammation of the bowels, as they are in fractured limbs.—p. 51.

In chronic peritonitis, "*A suspensory jacket*" is directed in addition to other curative means.—see page 52.

*Splenitis.* "The treatment is pretty much the same as in the preceding diseases; viz—cupping, leeching, mercurials, tartar-emetic, ointments, laxatives, *supporters* &c. p. 52.

*Strumous habit.*—"The remedies therefore that will be found best adapted to the first indication are *suitable suspensory jackets or an abdominal supporter* to prevent the viscera of the abdomen from gravitating from their natural position and to enable the patient to take proper exercise in the open air."—p. 75.

*Fe er.*—"If the fever arises from congestion, counter-irritants, friction, stimulants, revulsives, *bandages*, and dry cups of small size to put the fluids in motion are indicated."—p. 122. * * * "In others, it will require the use of *a roller applied to the extremities (so as to force a movement of the fluids in the superficial bloodvessels) assisted by* friction, bloodletting and warm brandy toddy to re-establish the circulation."—p. 125.

*Cholera.*—In addition to regulating "the secretions of the liver, stomach and bowels by the use of small and repeated doses of calomel and opium," we are directed to use "*bandages and supporters* to prevent gravitation of the viscera and to assist the capillary vessels in returning the blood to the heart."—p. 167.

"Those mechanical agents which I have found *absolutely necessary* to overcome congestion in cholera and check the profuse waste of the constituents of the blood by the morbid action of the skin and mucous membrane of the stomach and bowels, are as follows:—*the common muslin*

*roller for the extremities; a flannel roller for the abdomen; pledgets for the colon; small dry or wet cups on the surface of the body; the lancet, gentle friction aided by elevating the hips for a few minutes after the patient has been over the clo e stool.*"—Page 168–9.

*Hemorrhoids.*—"*The indication*" (*i. e.* to remove the exciting cause) "*may be met as follows: by inverting the body or elevating the hips, or by a recumbent posture on the right side, while the body slightly inclines forward, and the thighs are flexed to restore the sigmoid flexure to its proper position. After which a supporter should be applied with its left inguinal cushion about an inch thicker than its ordinary size. This additional fulness at its inner margin will prevent the sigmoid flexure from descending again into the true pelvic cavity, and thus remove one of the principal causes of piles.*"—Page 207.

We should doubtless convey to the reader a mistaken idea of Dr. Hood and his book, if we closed our notice of the volume here. It gives us sincere pleasure, however, to say, that amidst this mass of rubbish, there are some valuable jewels. The chapters on "Prolapsus Uteri" and "Gout" seem to us, in the main, very sensible and judicious—as indeed do all other parts of his work not taken up by his *discoveries* in pathology and practice. But these sound precepts form in reality so small a proportion of the whole, that careless readers might readily pass them by without discovering them. One thing may be said of the author, which, we are sure no one can fairly deny, and that is, that he is profoundly original in his conceptions of the nature and treatment of a multitude of diseases. But then there are many cases in which originality is not by any means to be commended; and we must say that we think this is one of them.

The concluding paragraph is so rich a *morceau* that we cannot refrain from giving it to our readers. Here it is:

"When the organization of the vital laboratory, contained within the abdomen and chest for the manufacture of the blood, and the laws of the animal functions, normal and deranged, are clearly understood; and when it is known what depurating organs should be checked or incited, and what are the therapeutical indications, which nature demands, then man need not be hurried off by functional derangement, but may live to the age allotted by his creator."

"Laugh and grow fat" is an old and profoundly philosophic maxim. We are sure that these extracts have added full fifteen pounds of adipose tissue to every one of our readers. We only hope that it has not settled upon their bowels and by its superadded weight induced "prolapsus of the abdominal viscera." R. J. B.

